# Effectiveness of imipenem-relebactam for multidrug-resistant *Pseudomonas aeruginosa* in pneumonia and bloodstream infections in the United States (MIRAGE)

**DOI:** 10.1128/aac.01325-25

**Published:** 2025-11-07

**Authors:** Walaiporn Wangchinda, Janet Y. Wu, Lilian M. Abbo, Renee Ackley, Patricia Bartley, Mayan Gilboa, Jeffrey Harrington, Rupal Jaffa, Megan E. Klatt, Ellen G. Kline, Ryan C. Kubat, Alexander J. Lepak, Erin K. McCreary, William R. Miller, Jeffrey C. Pearson, Sunish Shah, Truc T. Tran, Ana Vega, Melissa Vendetti, Emre Yucel, Jason M. Pogue, Ryan K. Shields

**Affiliations:** 1 University of Michigan, College of Pharmacy 15514, Ann Arbor, Michigan, USA; 2 Department of Pharmacy, Cleveland Clinic 2569, Cleveland, Ohio, USA; 3 Division of Infectious Diseases, Miller School of Medicine, University of Miami 698249, Miami, Florida, USA; 4 Deptarment of Infection Control & Antimicrobial Stewardship, Jackson Memorial Hospital 23215, Miami, Florida, USA; 5 Atrium Health 2351 , Charlotte, North Carolina, USA; 6 Department of Pharmacy, Kansas University Medical Center 21638, Kansas, Missouri, USA; 7 Department of Medicine, University of Pittsburgh 6614, Pittsburgh, Pennsylvania, USA; 8 University of Wisconsin, School of Medicine and Public Health 5232, Madison, Wisconsin, USA; 9 Division of Infectious Diseases, Houston Methodist Hospital 23534 , Houston, Texas, USA; 10 Department of Pharmacy, Brigham and Women’s Hospital, Boston, Massachusetts, USA; 11 University of Pittsburgh Medical Center 6619, Pittsburgh, Pennsylvania, USA; 12 Merck & Co Inc 2793 , Rahway, New Jersey, USA; Entasis, Big Bay, Michigan, USA

**Keywords:** imipenem-relebactam, *Pseudomonas*, pneumonia, resistance

## Abstract

Imipenem-relebactam demonstrates *in vitro* activity against multidrug-resistant (MDR) *Pseudomonas aeruginosa*, but real-world effectiveness data are limited. MIRAGE was a multicenter, retrospective, observational study of imipenem-relebactam for MDR *P. aeruginosa* pneumonia and bacteremia. Patients were included if they received imipenem-relebactam for >48 h within 7 days of index *P. aeruginosa* culture. The primary outcome was clinical success at day 30, defined as survival, resolution of signs and symptoms of infection, completion of intended treatment course, and absence of recurrent infection. Secondary outcomes included 30- and 90-day mortality, infection recurrence, and development of non-susceptibility. Sixty-three patients were included. Median (IQR) age was 61 (51–70) years, and the median Charlson Comorbidity Index was 5 (3–6). Forty-six percent of patients had an immunocompromising condition, 79% were in the intensive care unit, 76% were receiving mechanical ventilation, and 48% required vasopressors. Median SOFA score was 7 (5–12). Forty percent of index isolates that were tested displayed non-susceptibility to both ceftolozane-tazobactam and ceftazidime-avibactam. Fifty-six percent of patients achieved clinical success at day 30. All-cause 30- and 90-day mortality rates were 18% and 29%, respectively. Recurrent infections were documented in 37% of patients within 90 days, and resistance developed in 39% (16/41) of evaluable patients. Clinical outcomes following imipenem-relebactam for treatment of MDR *P. aeruginosa* were comparable to those reported in real-world studies for other novel β-lactam agents. Our data suggests that imipenem-relebactam has a role in the treatment of patients infected with MDR *P. aeruginosa*.

## INTRODUCTION

*Pseudomonas aeruginosa* is a leading cause of healthcare-associated infections, especially in critically ill and immunocompromised patients (1, 2). Clinical manifestations caused by *P. aeruginosa* are particularly concerning because of their high rates of mortality, recurrent infections, and development of antibiotic resistance (3). Infections due to multidrug-resistant (MDR) *P. aeruginosa* are associated with higher mortality rates than those caused by susceptible isolates (4). Recent US surveillance data indicate that 13–32% of *P. aeruginosa* isolates are categorized as MDR, 8–9% are extensively drug-resistant (XDR), and 7–8% exhibit the difficult-to-treat resistant (DTR) phenotype (5–11). Globally, the estimated annual deaths attributable to carbapenem-resistant *P. aeruginosa* increased from 30,200 in 1990 to 45,600 in 2021, underscoring an ongoing and significant public health concern (12).

Antibiotic resistance in *P. aeruginosa* evolves through multiple molecular mechanisms (13). Imipenem resistance is primarily mediated by loss of OprD porin channels in combination with hyperproduction of chromosomal AmpC β-lactamase (13). Relebactam is a non-β-lactam, bicyclic diazabicyclooctane β-lactamase inhibitor that inhibits class A and C β-lactamases (14). Among MDR *P. aeruginosa* isolates, the addition of relebactam restores antibacterial activity and lowers median imipenem MICs from 8 mg/L to 1 mg/L. Corresponding susceptibility rates increase from 24% to 85% (15). These *in vitro* data suggest a potential role of imipenem-relebactam as an effective treatment option for MDR *P. aeruginosa* infections.

The Infectious Diseases Society of America (IDSA) guidance document on treatment of antimicrobial-resistant Gram-negative infections endorses imipenem-relebactam, ceftolozane-tazobactam, and ceftazidime-avibactam as preferred treatment options for systemic infections caused by DTR *P. aeruginosa* (16). In clinical practice, ceftolozane-tazobactam and ceftazidime-avibactam are used more commonly given that they have been commercially available for over 10 years, which has resulted in more robust real-world data generation and adoption on automated susceptibility testing panels (17). Importantly, we previously reported that clinical use of these agents for MDR *P. aeruginosa* infections results in a high rate of recurrent infections and treatment-emergent resistance (18). Treatment-emergent resistance is mediated in part by point mutations, insertions, or deletions within the AmpC omega loop (19). Such mutations commonly confer cross-resistance to both agents but restore susceptibility to imipenem and imipenem-relebactam (20). Accordingly, imipenem-relebactam may represent a valuable therapeutic option in cases of treatment-emergent resistance to other novel β-lactams for *P. aeruginosa* infections.

Imipenem-relebactam efficacy was demonstrated in two phase-III multicenter randomized controlled trials—RESTORE-IMI 1 and 2—but these trials included a small number of patients with MDR *P. aeruginosa* infections (21, 22). Unfortunately, real-world evidence supporting the use of imipenem-relebactam for treatment of MDR *P. aeruginosa* infections remains limited. While some observational studies have been reported, the available data include a broad range of infection types, varying pathogens, and limited susceptibility testing results (23–27). To address this critical gap, MIRAGE was designed to evaluate the real-world efficacy of imipenem-relebactam specifically for the treatment of pneumonia and bloodstream infections caused by MDR *P. aeruginosa*.

## MATERIALS AND METHODS

### Study design and patient population

MIRAGE was a retrospective, multicenter, observational study conducted across nine healthcare centers in the United States. Eligible patients were adults (age ≥18 years) with pneumonia or bloodstream infections due to MDR *P. aeruginosa* from June 1, 2020, to January 31, 2025. Patients were eligible if the infecting *P. aeruginosa* isolate was non-susceptible to at least one agent in three or more antibiotic classes (28) and if they were treated with imipenem-relebactam for at least 48 h with treatment initiated within 7 days of index MDR *P. aeruginosa* isolate collection. Pneumonia was defined as the presence of a new or progressive infiltrate consistent with pneumonia on radiographic imaging with at least one of the following symptoms: purulent secretions, worsening cough or dyspnea, fever (≥38°C) or hypothermia (≤36°C), leukocytosis (≥10,000 white blood cells per µL), tachypnea (respiratory rate >22 breaths per minute), or increased oxygen requirements from baseline (29). Any type of respiratory culture was considered for the diagnosis. Bloodstream infections were defined as positive blood cultures yielding *P. aeruginosa*. Patients were excluded if they had osteomyelitis, septic arthritis, endocarditis, or empyema given that prolonged treatment durations and/or source control measures are needed for clinical response. Other exclusion criteria included cystic fibrosis; SARS-CoV-2 infection within the past 30 days; co-infection with carbapenem-resistant *Acinetobacter baumannii* (CRAb); or concurrent bloodstream infection with methicillin-resistant *Staphylococcus aureus* (MRSA). Patients were also excluded if the index *P. aeruginosa* isolate was non-susceptible to imipenem-relebactam based on the CLSI breakpoint (30); however, patient isolates that were not tested for imipenem-relebactam susceptibility were eligible.

### Data collection

Data were collated by site investigators from electronic health records into a standardized case report form (REDCap 14.3.3 Vanderbilt University). The University of Pittsburgh served as the coordinating center under local institutional review board number STUDY23080168. To ensure consistency in data entry, investigators were provided with a training video and a frequently asked questions document. Data inconsistencies and queries were addressed centrally with site investigators, and no missing data were identified after final adjudication. All case entries were reviewed by an independent principal investigator. Data collected included diagnosis, demographics, comorbid conditions, severity of illness, concomitant infections, antibiotic therapy, antimicrobial susceptibility testing results, and clinical outcomes. All documented indications for imipenem-relebactam initiation and discontinuation were also collected.

Immunosuppression was defined as having ≥1 of the following: neutropenia (absolute neutrophil count <1,500/µL); splenectomy; solid organ or bone marrow transplant; active malignancy requiring treatment; or receipt of high-dose corticosteroid (≥200 mg/day hydrocortisone or equivalent) for ≥2 weeks. Concomitant infections were defined as treatment of any non-*Pseudomonas* pathogens at any infection site during the course of study drug administration. Appropriate treatment for co-infecting pathogens was classified as receipt of an *in vitro* active agent targeting the pathogen(s). Combination therapy was defined as receipt of a second *in vitro* active antibiotic against *P. aeruginosa* for >48 h within the first 5 days of imipenem-relebactam treatment.

### Outcomes

The primary outcome was clinical success at day 30 from treatment initiation, defined as survival; resolution of signs and symptoms of infection; completion of the intended treatment duration without the need for extension due to protracted clinical response or antibiotic escalation due to worsening infection, development of resistance, or toxicity; and absence of recurrent infection due to MDR *P. aeruginosa*. Secondary outcomes included clinical success at day 7 (defined similarly to day 30 but not requiring complete resolution of signs and symptoms), all-cause 30- and 90-day mortality, recurrent infections, and the development of resistance to imipenem-relebactam within 90 days from treatment onset. Length of stay, discharge disposition, and adverse events were also collected. The development of imipenem-relebactam resistance was defined as isolation of any subsequent isolate demonstrating non-susceptibility to imipenem-relebactam after a minimum of 72 h of treatment. Susceptibility testing was conducted by microbiology laboratories according to local practices, and results were retrieved retrospectively (31). Only patients with documented baseline susceptibility to imipenem-relebactam were included in the assessment of resistance development.

## RESULTS

### Description of the cohort

Of 255 patients who received imipenem-relebactam for >48 h at study sites, a total of 63 patients were included. The reasons for exclusion were use of imipenem-relebactam for a non-eligible *P. aeruginosa* infection type (*n* = 65), cystic fibrosis (*n* = 38), documented imipenem-relebactam non-susceptibility (*n* = 22), metastatic infection requiring prolonged treatment (*n* = 19), respiratory colonization (*n* = 13), co-infection with SARS-CoV-2 (*n* = 11), treatment initiation >7 days after index MDR *P. aeruginosa* culture (*n* = 11), co-infection with CRAb or MRSA bacteremia (*n* = 10), or other reasons (*n* = 3). The median age of included patients was 61 years (IQR 51–70); 64% (40/63) were male; and 67% (42/63) were White (Table 1). The median Charlson Comorbidity Index (CCI) and Sequential Organ Failure Assessment (SOFA) scores were 5 (IQR 3–6) and 7 (IQR 5–12), respectively. Overall, 79% (50/63) of patients were managed in the intensive care unit (ICU), 76% (48/63) required invasive mechanical ventilation, 35% (22/63) renal replacement therapy, and 48% (30/63) vasopressor support. At least one immunocompromising condition was identified in 46% (29/63) of patients. Pneumonia was the predominant infection in 87% (55/63) of patients and was further categorized as ventilator-associated, ventilated hospital-acquired, community-acquired, or hospital-acquired in 69%, 11%, 11%, and 9%, respectively.

**TABLE 1:** Baseline demographics and clinical characteristics of patients treated with imipenem-relebactam for MDR *P. aeruginosa* infections^*b*^

Variable	*N* = 63
Median age in years (IQR)	61 (51–70)
Male sex, *n* (%)	40 (64)
Race, *n* (%)	
White	42 (67)
Black/African American	12 (19)
Other	9 (14)
Median Charlson Comorbidity Index (IQR)	5 (3–6)
Median SOFA score (IQR)	7 (5–12)
Origin of hospital admission, *n* (%)	
Home	33 (52)
Outside hospital	17 (27)
LTAC/SNF	11 (18)
Inpatient rehab	2 (3)
ICU at treatment initiation, *n* (%)	50 (79)
Mechanical ventilation, *n* (%)	48 (76)
On chronic mechanical ventilation at the time of admission	31 (49)
Receipt of renal replacement therapy, *n* (%)	22 (35)
Severe sepsis/septic shock, *n* (%)	45 (71)
Receipt of vasopressors, *n* (%)	30 (48)
Any immunocompromising condition, *n* (%)	29 (46)
Solid-organ transplant recipient	21 (33)
Liver	7 (11)
Heart	6 (10)
Lung	6 (10)
Other immunocompromising condition	11 (18)
Chronic steroid use	8 (13)
Active solid malignancy	4 (6)
Bone marrow transplant	1 (2)
ANC <1,500 cells/µL	1 (2)
Splenectomy	1 (2)
Bacteremia, *n* (%)	8 (13)
Pneumonia, *n* (%)	55 (87)
Ventilator-associated	38 (69);
Ventilated hospital-acquired	6 (11)
Community-acquired	6 (11)
Hospital-acquired	5 (9)
Use of any novel β-lactam agents within 12 months, *n* (%)	34 (54)
Ceftolozane-tazobactam	20 (32)
Ceftazidime-avibactam	17 (27)
Cefiderocol	7 (11)
Imipenem-relebactam	5 (8)
Index isolate susceptibility to imipenem/relebactam, *n* (%)^*a*^	
>22 mm by disk diffusion	3 (5)
≤2 mg/L by gradient strip testing	15 (24)
≤2 mg/L broth microdilution	23 (37)
Baseline MIC = 0.5 mg/L	5 (8)
Baseline MIC = 1 mg/L	15 (24)
Baseline MIC = 2 mg/L	18 (29)
Baseline MDR *P. aeruginosa* isolate not tested	22 (35)
Baseline non-susceptibility to alternative β-lactam, n/isolate tested (%)	
Ceftolozane/tazobactam	25/52 (48)
Ceftazidime/avibactam	29/48 (60)
Ceftolozane/tazobactam and ceftazidime/avibactam	18/45 (40)
Cefiderocol	1/15 (7)

^*a*^ Additional details on susceptibility testing methods and results are provided in Table S1.

^*b*^ ANC, absolute neutrophil count; ICU, intensive care unit; IQR, interquartile range; LTAC, long-term acute care facility; MDR, multidrug-resistant; SNF, skilled nursing facility; SOFA, sequential organ failure assessment.

At the time of index infection, 54% (34/63) of patients had received another novel β-lactam within the 12 months preceding imipenem-relebactam initiation. Antimicrobial susceptibility testing of index isolates to imipenem-relebactam was performed in 65% (41/63) of cases (Table 1). The modal baseline imipenem-relebactam MIC was 2 mg/L (range: 0.5–2 mg/L). Baseline MDR *P. aeruginosa* isolates from three patients were tested by disk diffusion. Non-susceptibility rates for ceftolozane-tazobactam and ceftazidime-avibactam against index *P. aeruginosa* isolates were 48% (25/52) and 60% (29/48), respectively (Table S1).

### Treatment characteristics** **

The most common indication for imipenem-relebactam initiation was reported susceptibility results, either for the index infection or due to known resistance in prior *P. aeruginosa* isolates (*n* = 49; Table 2). Other indications included ceftolozane-tazobactam unavailability (*n* = 18), the presence of co-infecting pathogens (*n* = 8), and clinical failure of a prior antibiotic regimen for MDR *P. aeruginosa* (*n* = 6). The median time from index culture to initiation of imipenem-relebactam was 67 hours (IQR 25–98), with 40% (25/63) of patients receiving the first dose of imipenem-relebactam within 48 h of index culture collection. Combination therapy with imipenem-relebactam was administered in 25% (16/63) of patients, most commonly with adjunctive aerosolized tobramycin. Co-infection was present in 33% (21/63) of patients, all of whom received appropriate treatment. The total median duration of imipenem-relebactam was 8 days (IQR 5–13), and 64% (40/63) of patients completed therapy as planned.

**TABLE 2:** Characteristics of real-world imipenem/relebactam use in pneumonia and bloodstream infections^*a*^

Treatment characteristics	*N* = 63
Indication for imipenem/relebactam use, *n* (%)	
MDR P. aeruginosa susceptibility results	49 (78)
Ceftolozane/tazobactam was not available	18 (29)
Presence of co-infecting organisms^*b*^	8 (13)
Failing prior regimen	6 (10)
Previous exposure to ceftazidime/avibactam or ceftolozane/tazobactam	2 (3)
Median time to imipenem/relebactam, hours (IQR)	67 (25–98)
Active treatment before study drug, *n* (%)	16 (25)
Receipt of prolonged infusion^*c*^, *n* (%)	1 (2)
Combination treatment, *n* (%)	16 (25)
Inhaled antibiotics	11 (69)
Intravenous antibiotics	3 (19)
Both inhaled and intravenous	2 (12)
Co-infection, *n* (%)	21 (33)
Co-infection appropriately treated	21 (100)
Median treatment duration in days (IQR)	8 (5–13)
Reason for imipenem/relebactam discontinuation, *n* (%)	
Completed course as planned	40 (64)
De-escalation based on susceptibility results	8 (13)
Transfer to CMO/hospice or death	5 (8)
Antibiotics modified due to worsening infection	4 (6)
Discharged prior to end of treatment	2 (3)
Toxicity	1 (2)
Other	3 (5)

^*a*^ CMO, comfort measures only; IQR, interquartile range; MDR, multidrug-resistant.

^*b*^ Co-infecting organisms included *Enterobacterales *(*n *= 6)*, Enterococcus *(*n *= 1)*, *and *Mycobacterium abscessus *(*n *= 1).

^*c*^ Prolonged infusions were defined as infusion times ≥3 h.

### Outcomes

**Fig 1:**
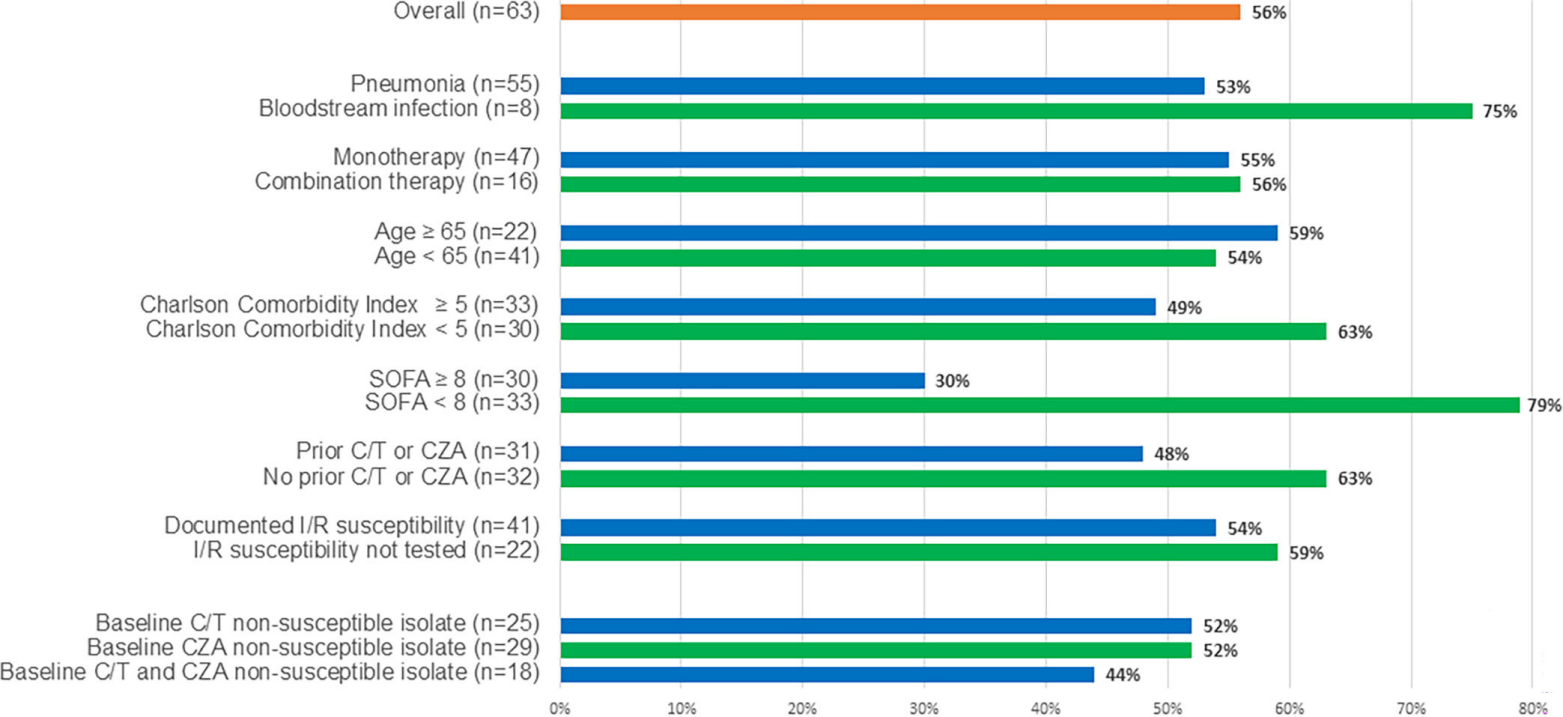
Primary outcome of clinical success at day 30 following treatment with imipenem-relebactam across patient subgroups. C/T, ceftolozane-tazobactam; CZA, ceftazidime-avibactam; I/R, imipenem-relebactam; SOFA, sequential organ failure assessment. n = number of patients in subgroup.

The primary outcome of clinical success at day 30 was achieved in 56% (35/63) of patients. Among the 28 patients who experienced treatment failure, the reasons for failure were recurrent infection in 22% (14/63), death in 18% (11/63), and/or lack of clinical improvement in 14% (9/63). Rates of clinical success by subgroup are shown in Fig. 1.

**Fig 2:**
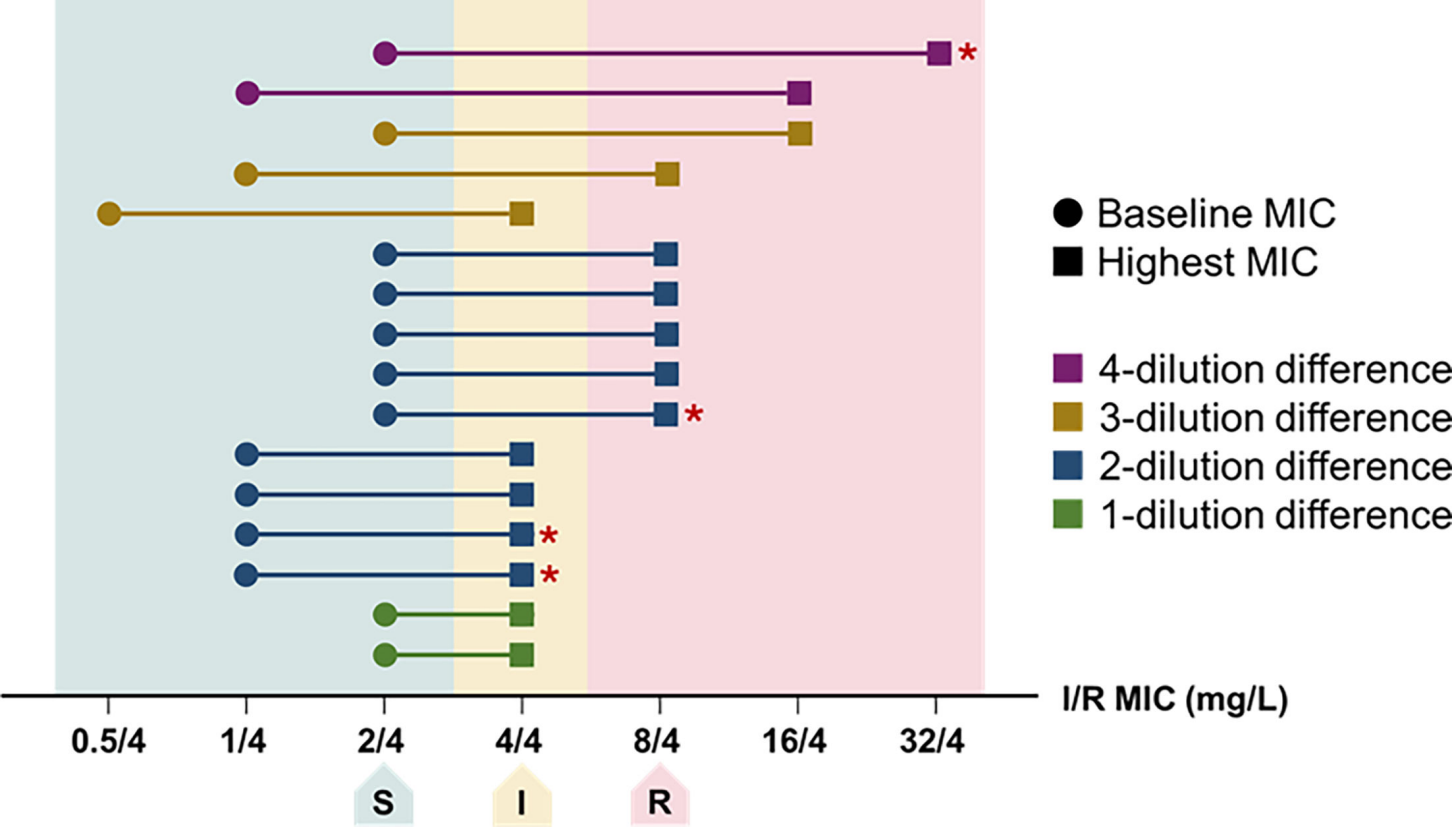
Imipenem-relebactam MIC changes across patients with evidence of treatment-emergent non-susceptibility within 90 days. I, intermediate; I/R, imipenem-relebactam; MIC, minimum inhibitory concentration; R, resistant; S, susceptible. * denotes patients with isolates that later reverted to imipenem/relebactam-susceptible. Each horizontal line represents the baseline isolate and subsequent isolate with the highest post-treatment MIC by unique patient.

Clinical success at day 7 was observed in 79% (50/63) of patients (Table 3). All-cause 30- and 90-day mortality rates were 18% (11/63) and 29% (18/63), respectively. The median total length of hospital stay was 63 days (IQR 23–121), including a median of 27 days (IQR 13–66) after the initiation of imipenem-relebactam. Among index isolates exhibiting baseline susceptibility to imipenem-relebactam, subsequent resistance was documented in 39% (16/41). Notably, imipenem-relebactam MICs increased by ≤4-fold in 69% (11/16) of paired susceptible and resistant isolates. Non-susceptible isolates were categorized as intermediate or resistant in 44% (7/16) and 56% (9/16) of instances, respectively (Fig. 2). Restored imipenem-relebactam susceptibility following identification of non-susceptibility was noted in 25% (4/16) of cases after treatment discontinuation. In two resistance cases, a second treatment course of imipenem-relebactam was initiated for recurrent infections prior to the development of resistance. One patient developed interstitial nephritis, which was considered to be related to imipenem-relebactam administration. No other adverse events were reported.

**TABLE 3:** Clinical outcomes of patients treated with imipenem/relebactam for MDR *P. aeruginosa* pneumonia or bacteremia^*a*^

Clinical outcomes	*N* = 63
Clinical success at day 7, *n* (%)	50 (79)
Clinical success at day 30, *n* (%)	35 (56)
All-cause mortality at day 30, *n* (%)	11 (18)
All-cause mortality at day 90, *n* (%)	18 (29)
Recurrent infection within 30 days, *n* (%)	14 (22)
Recurrent infection within 90 days, *n* (%)	23 (37)
Median time to recurrent infection from treatment initiation in days (IQR)	31 (18–49)
Development of resistance within 30 days, *n* (%)	12/41 (29)
Development of resistance within 90 days, *n* (%)	16/41 (39)
Median time to resistance from treatment initiation in days (IQR)	12 (8–48)
Median total hospital length of stay in days (IQR)	63 (23–121)
From time of treatment start in days (IQR)	27 (13 – 66)
Disposition at hospital discharge, *n* (%)	
LTAC/SNF/rehab	27 (43)
Home	17 (27)
Death/hospice	17 (27)
Outside hospital	2 (3)

^*a*^ IQR, interquartile range; LTAC, long-term acute care facility; MDR, multidrug-resistant; SNF, skilled nursing facility.

## DISCUSSION

This multicenter study provides important real-world evidence for the efficacy of imipenem-relebactam in the treatment of MDR *P. aeruginosa* pneumonia and bloodstream infections among critically ill patients. The rate of 30-day clinical success following imipenem-relebactam treatment was 56%, which is closely aligned with previously reported rates of success for ceftolozane-tazobactam (61%) and ceftazidime-avibactam (52%) in a comparable patient population (18). Likewise, the rates of 30- and 90-day mortality (18% and 29%, respectively) were similar to those documented following treatment with ceftolozane-tazobactam (23% and 38%, respectively) or ceftazidime-avibactam (24% and 37%, respectively) in the aforementioned study. Taken together, the results of the MIRAGE study support the role of imipenem-relebactam as a viable treatment option for MDR *P. aeruginosa* infections.

These data also provide critical insights into the usage patterns for imipenem-relebactam in the United States. Over half of the index isolates demonstrated resistance to ceftolozane-tazobactam or ceftazidime-avibactam. Not surprisingly, more than half of patients had previous exposure to ≥1 of these agents or cefiderocol. The cumulative data suggest that imipenem-relebactam is often reserved for treatment of *P. aeruginosa* isolates with documented resistance to other novel β-lactams. This approach is in line with the previously described collateral susceptibility of imipenem-relebactam against MDR *P. aeruginosa* treated with ceftolozane-tazobactam or ceftazidime-avibactam (19, 20). Comparable rates of clinical success demonstrated in MIRAGE suggest that imipenem-relebactam can be a reliable agent in this challenging clinical setting. Next, the overall number of real-world cases reported in MIRAGE is numerically lower than those previously reported for ceftolozane-tazobactam or ceftazidime-avibactam in the United States (18, 19, 32). This reflects a lower usage rate compared to other novel β-lactams (33), but also highlights the stringent inclusion criteria applied. Prior studies have included more patients treated with imipenem-relebactam than the current analysis (23); however, the heterogeneity of infection types, patient populations, pathogens, and susceptibility results makes drawing conclusions particularly challenging.

Our patient population demonstrated a potentially concerning trend in the emergence of imipenem-relebactam resistance following treatment. The overall rate of emergent non-susceptibility was 39% within 90 days of treatment initiation, which is higher than the rates we previously reported for ceftolozane-tazobactam or ceftazidime-avibactam using the same criteria (18). There are, however, several important caveats to consider in interpreting these data. First, the methods used to evaluate susceptibility to imipenem-relebactam varied across centers and included the use of gradient testing strips that were subsequently recalled by the FDA due to poor performance and the identification of false resistance (34). In our study, 25% (4/16) resistance cases were identified using gradient strip testing (Table S1). Secondly, the modal MIC for imipenem-relebactam against MDR *P. aeruginosa,* both in large United States surveillance studies and the current study, is 2 mg/L (11) (Table 1), which is the CLSI susceptibility breakpoint (30). Given that the majority of resistance cases were associated with a ≤4-fold MIC increase, we hypothesize that the variability in MIC testing may explain the higher rate of resistance. In accordance with this hypothesis, 25% of non-susceptible cases were associated with subsequent isolation of imipenem-relebactam–susceptible *P. aeruginosa* within the 90-day analysis timeframe. Finally, a high proportion of patients in the MIRAGE study had previously been treated with other novel β-lactams, which was not the case in our prior CACTUS study (18). This likely reflects treatment of refractory infections due to MDR *P. aeruginosa* that may overexpress AmpC β-lactamases and/or transmembrane efflux pumps that provide a first step toward imipenem-relebactam resistance (20, 31). Future studies are needed to determine the potential impact of imipenem-relebactam resistance against *P. aeruginosa* on clinical outcomes, the predominant molecular mechanisms of resistance, and the non-susceptibility rates measured by standardized testing in a central laboratory.

While the MIRAGE study represents a significant advance in the real-world evidence for imipenem-relebactam, several limitations should be recognized. First, despite collecting data across nine study sites over 5 years, the sample size of the study is relatively small. These data reflect the current usage of imipenem-relebactam in the United States, which is highly dependent upon its position within hospital formularies and access to reliable susceptibility testing methods in clinical practice. In fact, a total of 19 additional centers were approached for participation in the study but were not included because too few cases met inclusion criteria or imipenem-relebactam had not been added to their hospital formulary. Our inclusion and exclusion criteria were also intentionally strict, resulting in <25% of patients who received imipenem-relebactam for MDR *P. aeruginosa* cultures being included in the study. Therefore, while the findings report robust clinical outcomes in a medically complex, high-risk patient cohort with definitive infections, the trade-off is a modest sample size. Additionally, the study was conducted retrospectively, which introduces the potential for bias, including confounding by indication. Next, patients were included if infections were due to MDR *P*. aeruginosa; however, we did not collect complete antibiotic susceptibility data to confirm which specific agents were reported as resistant. Finally, the lack of a comparator group limits the ability to directly assess the relative effectiveness of imipenem-relebactam compared to other treatment options, which the current study was not designed to do.

In conclusion, in this critically ill patient population, we found that imipenem-relebactam was often used following treatment with other novel β-lactams for the treatment of MDR *P. aeruginosa* infections. Clinical outcomes were generally comparable to those previously reported in real-world studies for alternative novel β-lactam agents. These data demonstrate that imipenem-relebactam plays an important role in the treatment of MDR *P. aeruginosa* infections, even when other agents are not available or demonstrate *in vitro* resistance. However, further comparative data are needed to define the precise role of imipenem-relebactam for the treatment of *P. aeruginosa* infections.

## SUPPLEMENTAL MATERIAL

Table S1 aac.01325-25-s0001.xlsx Details of MIC testing.

ASM does not own the copyrights to Supplemental Material that may be linked to, or accessed through, an article. The authors have granted ASM a non-exclusive, world-wide license to publish the Supplemental Material files. Please contact the corresponding author directly for reuse.
